# Supplementary material for: Heterogeneity in quiescent Müller glia in the uninjured zebrafish retina drive differential responses following photoreceptor ablation
Source: Front Mol Neurosci. 2023 Jul 27;16:1087136. doi: 10.3389/fnmol.2023.1087136 (PMC10413128; doi:10.3389/fnmol.2023.1087136)
Supplement: Supplementary file 1 [file Image_1.pdf]

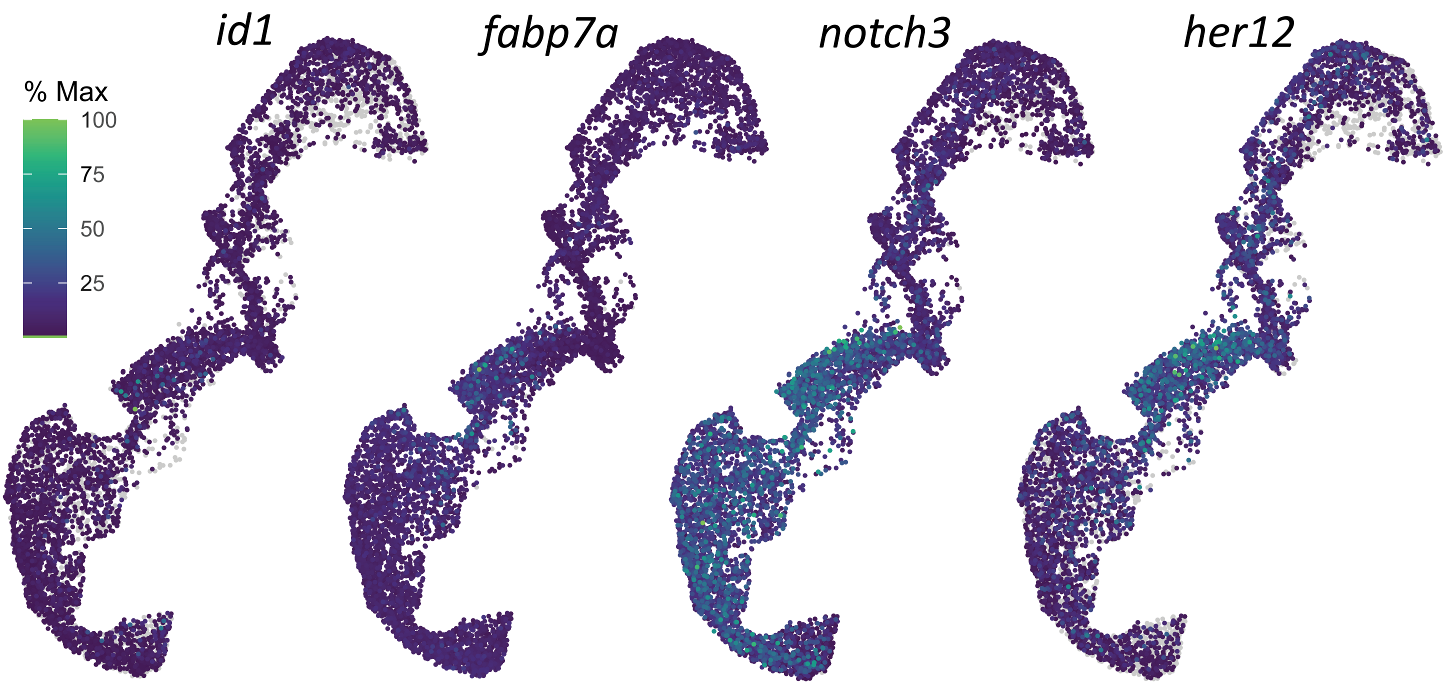

Supplementary Figure 1: id1, fabp7a, notch3 and her12 expression in Müller glia along pseudotime in integrated Lws2 and Sws2 photoreceptor ablation scRNA-seq datasets.
