# Supplementary figures and images for: Heterogeneity in quiescent Müller glia in the uninjured zebrafish retina drive differential responses following photoreceptor ablation
Source: Front Mol Neurosci. 2023 Jul 27;16:1087136. doi: 10.3389/fnmol.2023.1087136 (PMC10413128; doi:10.3389/fnmol.2023.1087136)

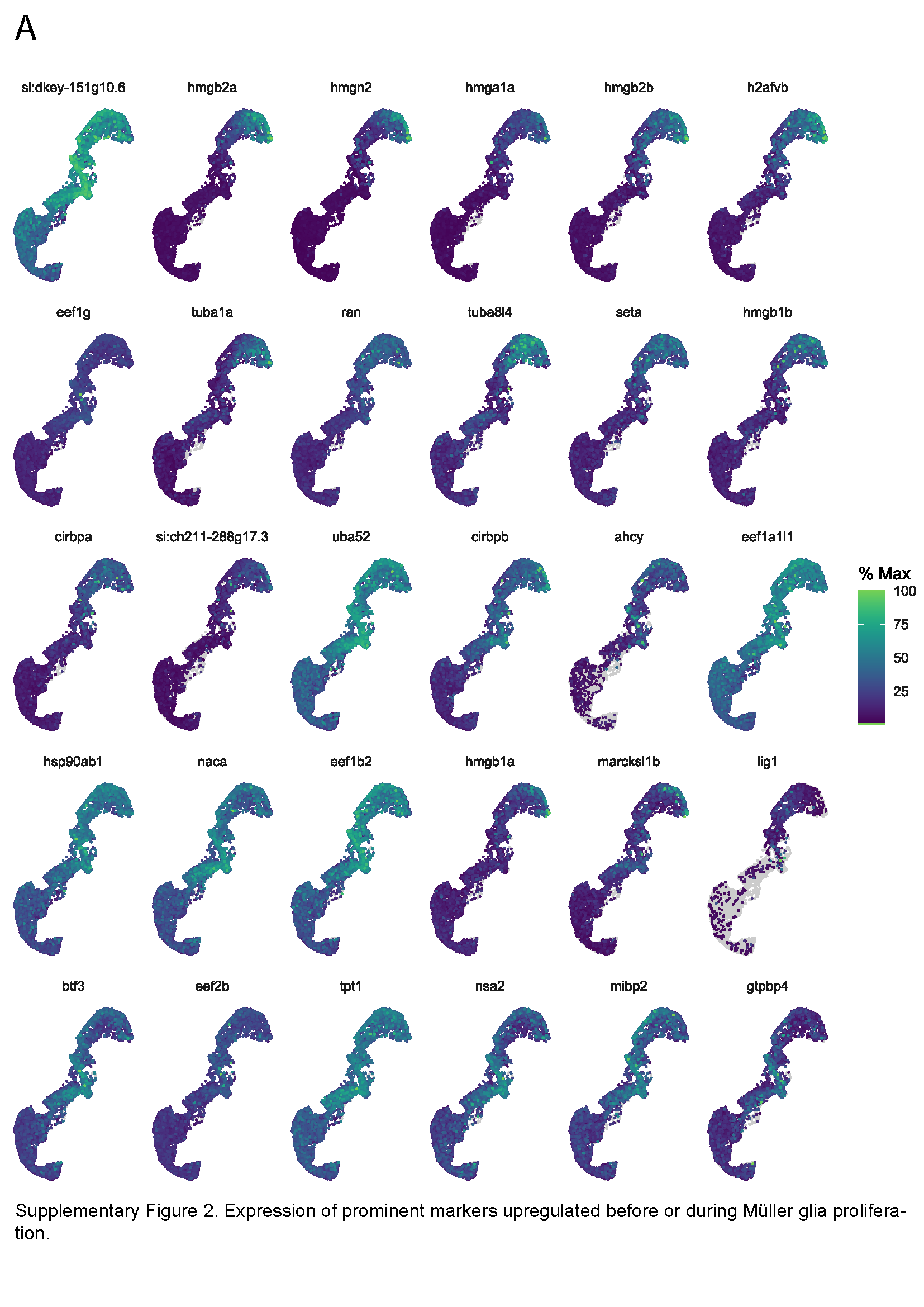

Supplement: Supplementary file 2 [file Image_2.tiff]
