# Supplementary material for: Heterogeneity in quiescent Müller glia in the uninjured zebrafish retina drive differential responses following photoreceptor ablation
Source: Front Mol Neurosci. 2023 Jul 27;16:1087136. doi: 10.3389/fnmol.2023.1087136 (PMC10413128; doi:10.3389/fnmol.2023.1087136)
Supplement: Supplementary file 3 [file Image_3.pdf]

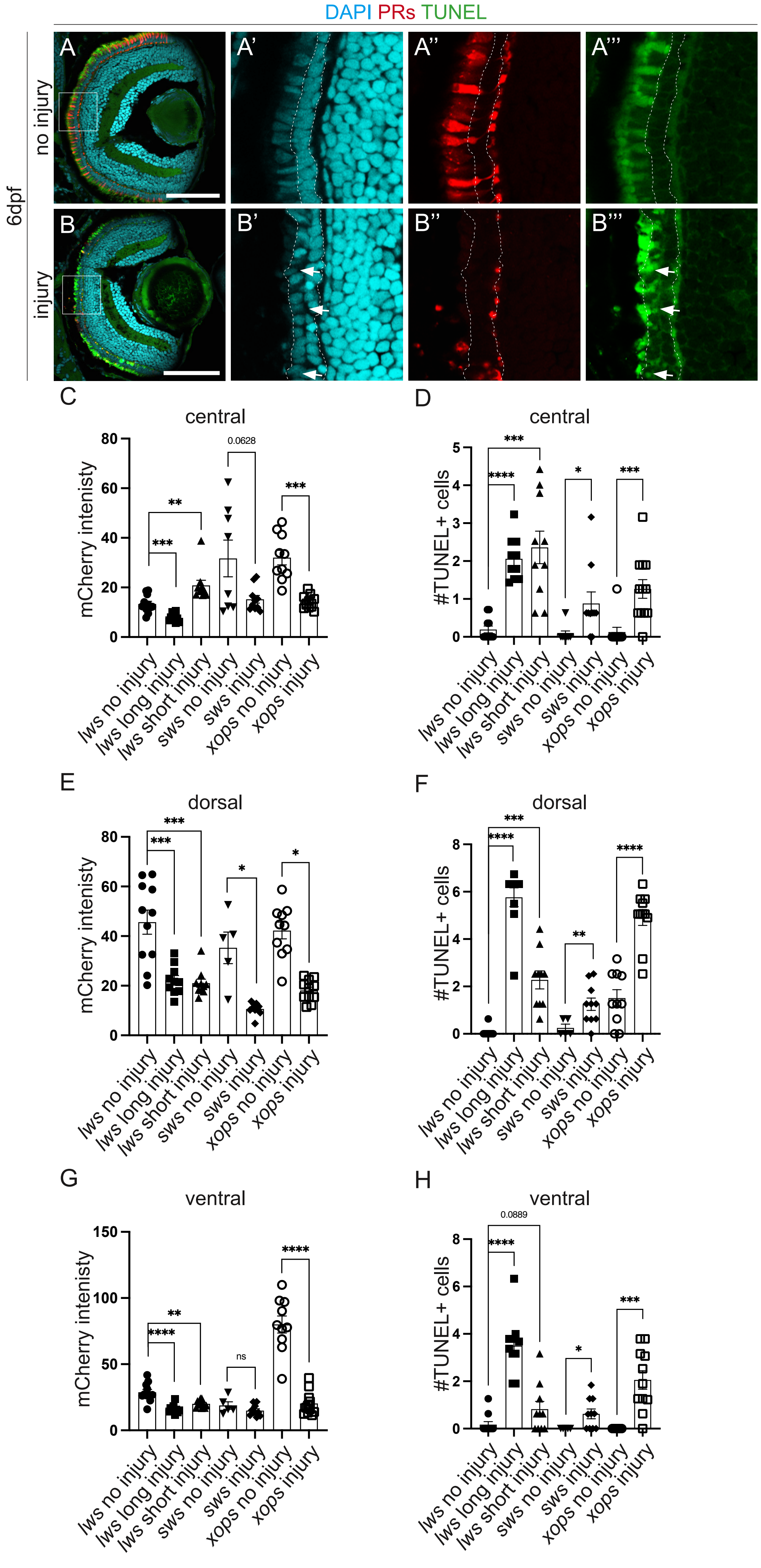

Supplementary Figure 3: Characteristic spatial cell ablation in each of the injury models. Micrographs in A (no injury control) and B (injury at 4 dpf) show loss of mCherry label in the targeted photoreceptors (Lwsw in this example) and increased TUNEL labelled cells in the outer nuclear layer (ONL dotted lines), which contains photoreceptor cell bodies. For each of the four different ablation paradigms (Lws2 large injury, Lws2 small injury, Sws2 injury and Xops injury) 2 days following injury (at 6 dpf), mCherry signal was reduced and TUNEL labelled dying cells in the ONL were increased across central, dorsal and ventral regions of the retina. There were some spatial differences related to the relative abundance of Lws2, Sws2 and Xops labelled photoreceptors in these regions. Scale bar = 50 μm.
