# Supplementary material for: Heterogeneity in quiescent Müller glia in the uninjured zebrafish retina drive differential responses following photoreceptor ablation
Source: Front Mol Neurosci. 2023 Jul 27;16:1087136. doi: 10.3389/fnmol.2023.1087136 (PMC10413128; doi:10.3389/fnmol.2023.1087136)
Supplement: Supplementary file 5 [file Image_5.pdf]

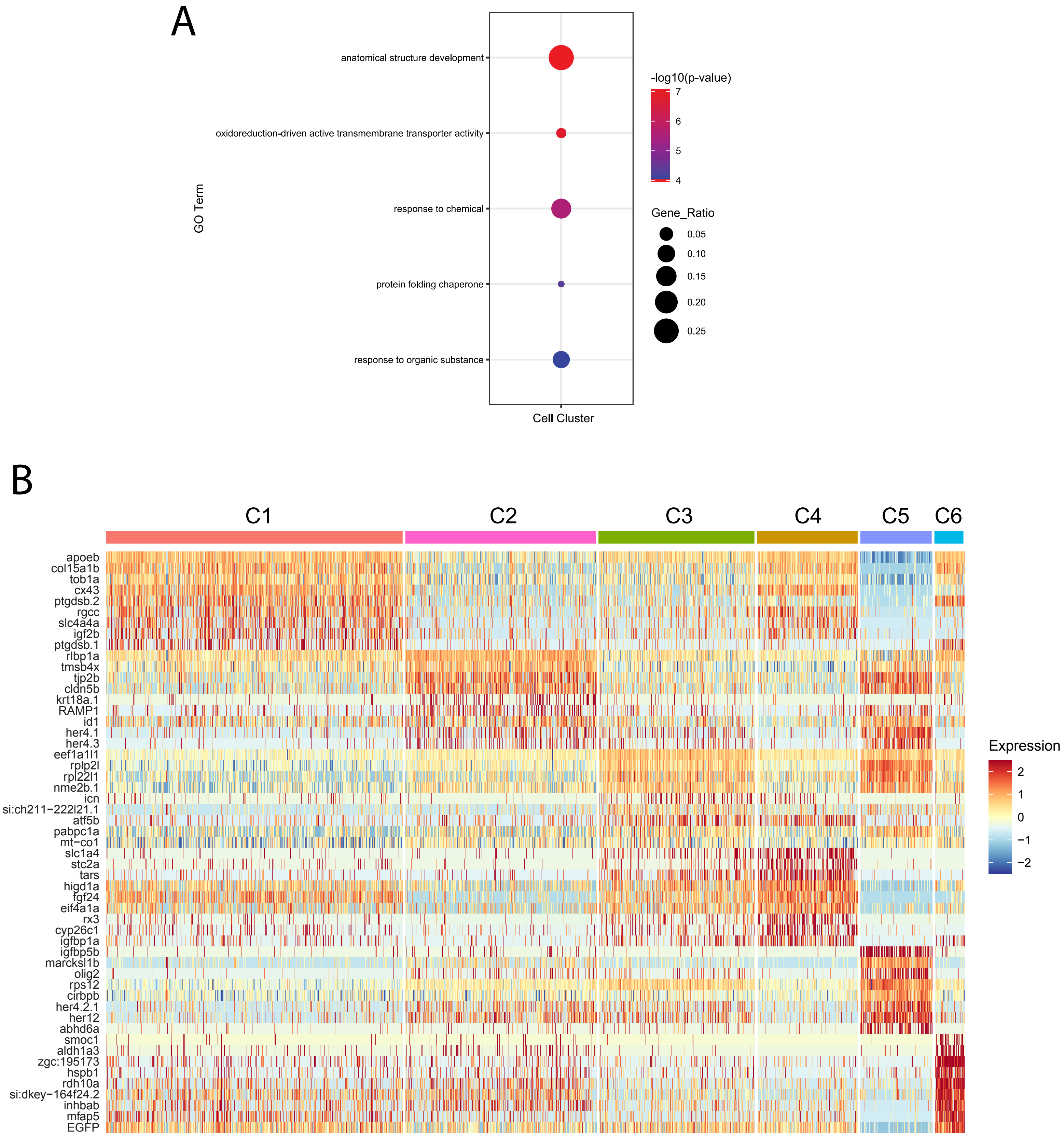

Supplementary Figure 5: (A) Enrichment term analysis of the Müller glia cluster expressing markers associated with stress response. (B) Heatmap visualizing the top 10 expressed genes of each quiescent Müller glia cluster (C1-C6).
