# Supplementary material for: Heterogeneity in quiescent Müller glia in the uninjured zebrafish retina drive differential responses following photoreceptor ablation
Source: Front Mol Neurosci. 2023 Jul 27;16:1087136. doi: 10.3389/fnmol.2023.1087136 (PMC10413128; doi:10.3389/fnmol.2023.1087136)
Supplement: Supplementary file 6 [file Image_6.pdf]

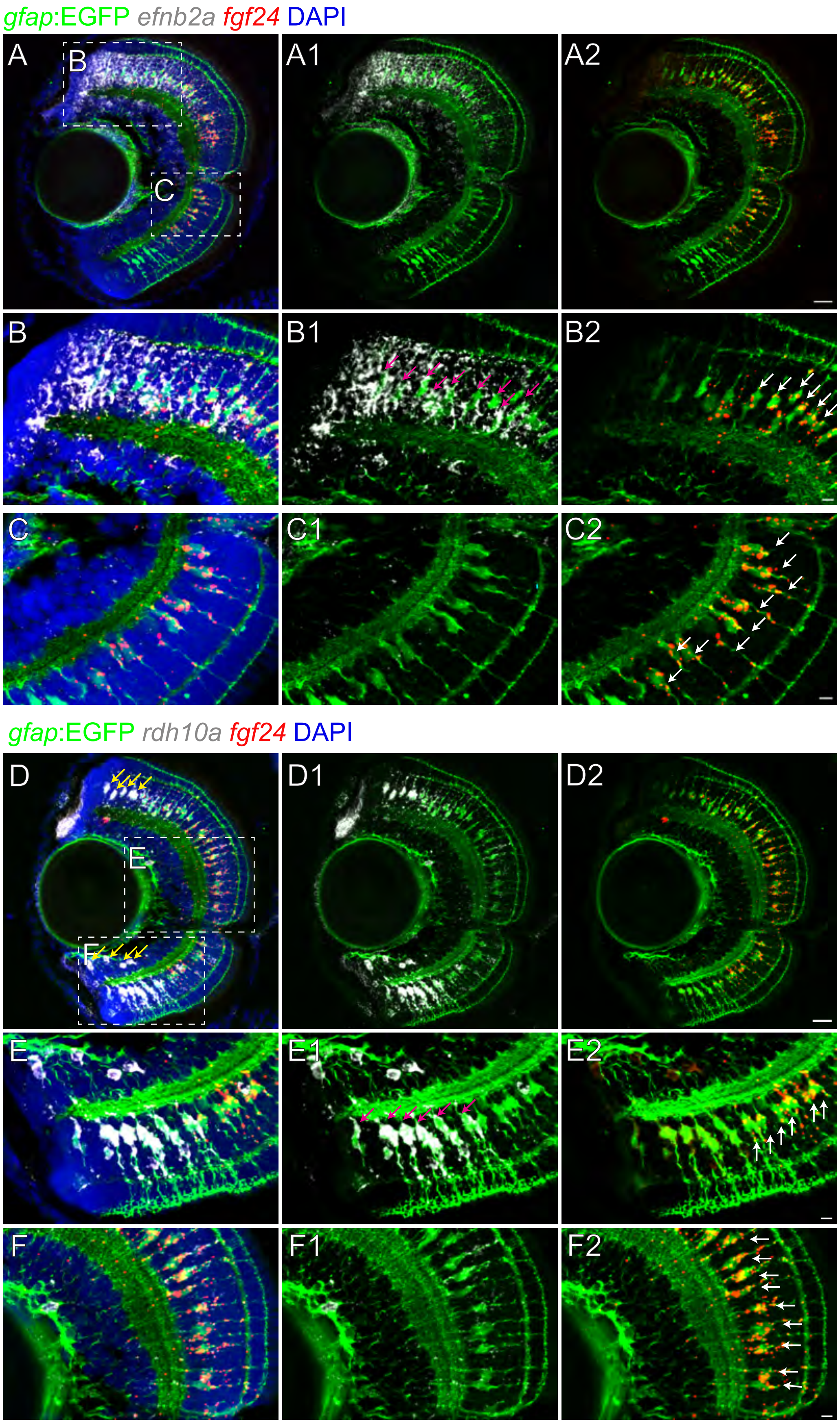

Supplementary Figure 6. (A-F) RNAscope *in situ hybridization* reveals the colocalization of *efnb2a* (magenta arrows in B1) *fgf24* (white arrows in B2, C2, E2 and F2), and *rdh10a* (magenta arrows in E1) with dorsal, central, and ventral gfap:EGFP+ Müller glia, respectively. A small subset of Müller glia in the dorsal retina and non-MG in ventral retina (yellow arrows in D) also express *rhd10a* (D-D2). Scale bars A-A2, D-D2 = 20 μm; B-C2, E-F2 = 5 μm.
