# Supplementary material for: Heterogeneity in quiescent Müller glia in the uninjured zebrafish retina drive differential responses following photoreceptor ablation
Source: Front Mol Neurosci. 2023 Jul 27;16:1087136. doi: 10.3389/fnmol.2023.1087136 (PMC10413128; doi:10.3389/fnmol.2023.1087136)
Supplement: Supplementary file 7 [file Image_7.pdf]

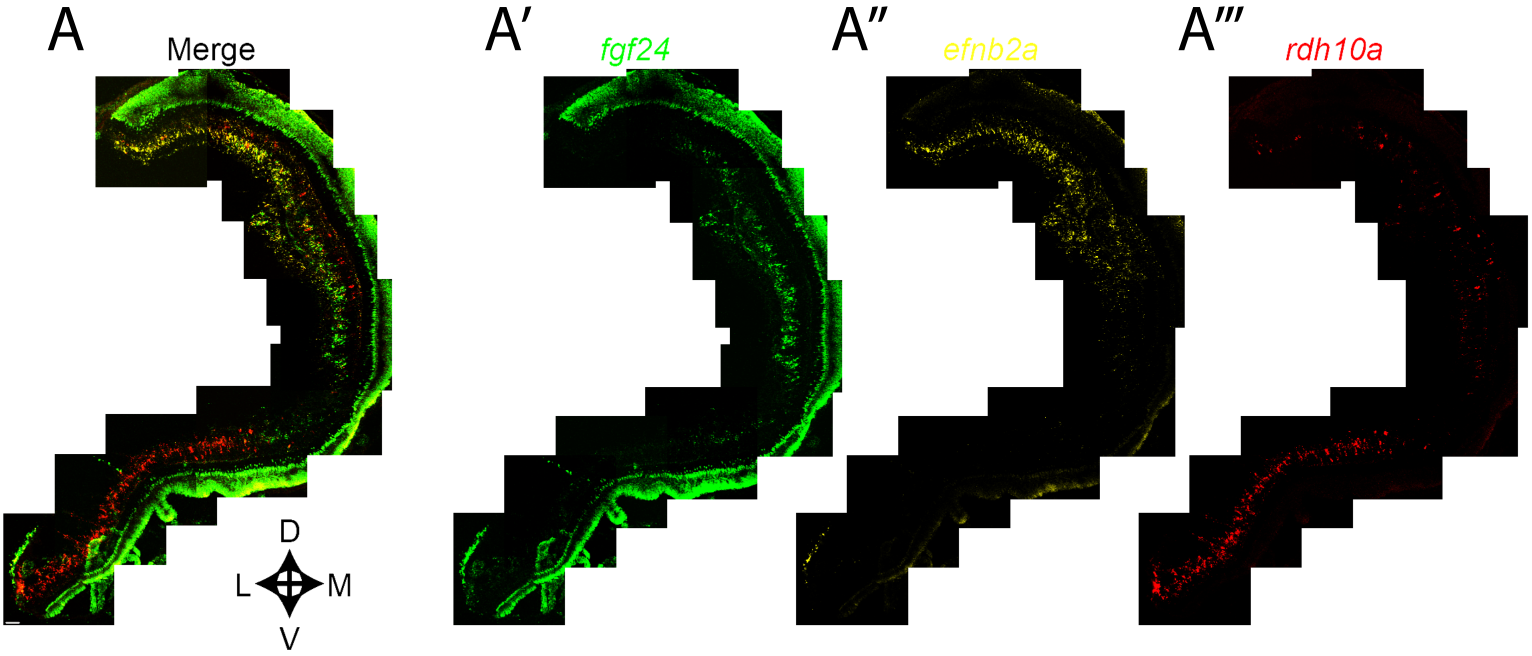

(A) RNAscope in situ hybridization of markers fgf24 (A'), efnb2a (A'') and rdh10a (A''') in the 12 month-old zebrafish retina.
