# Supplementary material for: Heterogeneity in quiescent Müller glia in the uninjured zebrafish retina drive differential responses following photoreceptor ablation
Source: Front Mol Neurosci. 2023 Jul 27;16:1087136. doi: 10.3389/fnmol.2023.1087136 (PMC10413128; doi:10.3389/fnmol.2023.1087136)
Supplement: Supplementary file 8 [file Image_8.pdf]

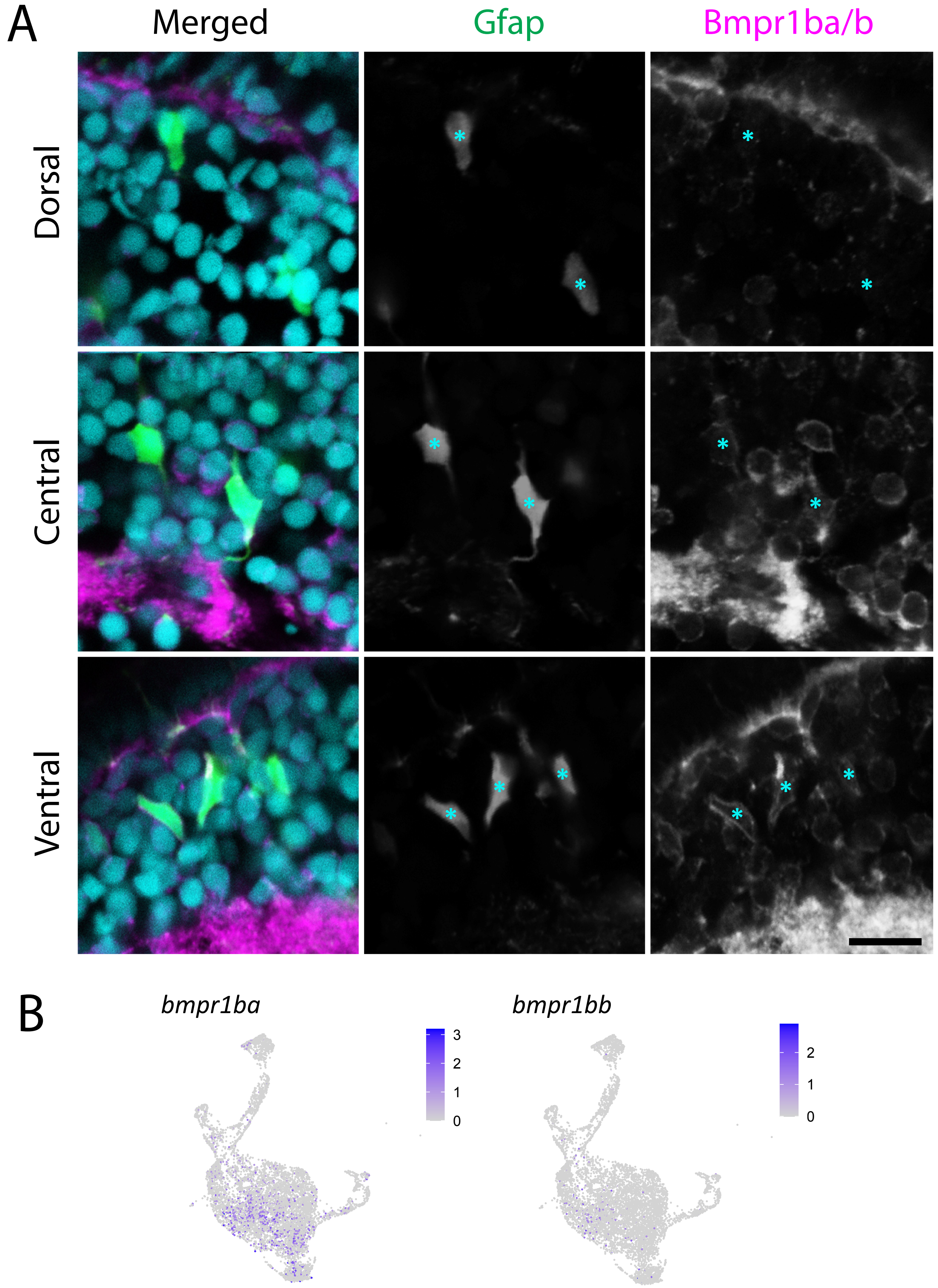

Supplementary Figure 7: (A) Labelling of DAPI-positive nuclei (cyan), Gfap-expressing Müller glia and Bmpr1ba/b. Asteriks indicate cell bodies of Müller glia in dorsal, central and ventral regions of the retina. Scale Bar = 10 µm. (B) Expression plots of the Bmpr1ba/b encoding genes.
