# Supplementary material for: Heterogeneity in quiescent Müller glia in the uninjured zebrafish retina drive differential responses following photoreceptor ablation
Source: Front Mol Neurosci. 2023 Jul 27;16:1087136. doi: 10.3389/fnmol.2023.1087136 (PMC10413128; doi:10.3389/fnmol.2023.1087136)
Supplement: Supplementary file 9 [file Image_9.pdf]

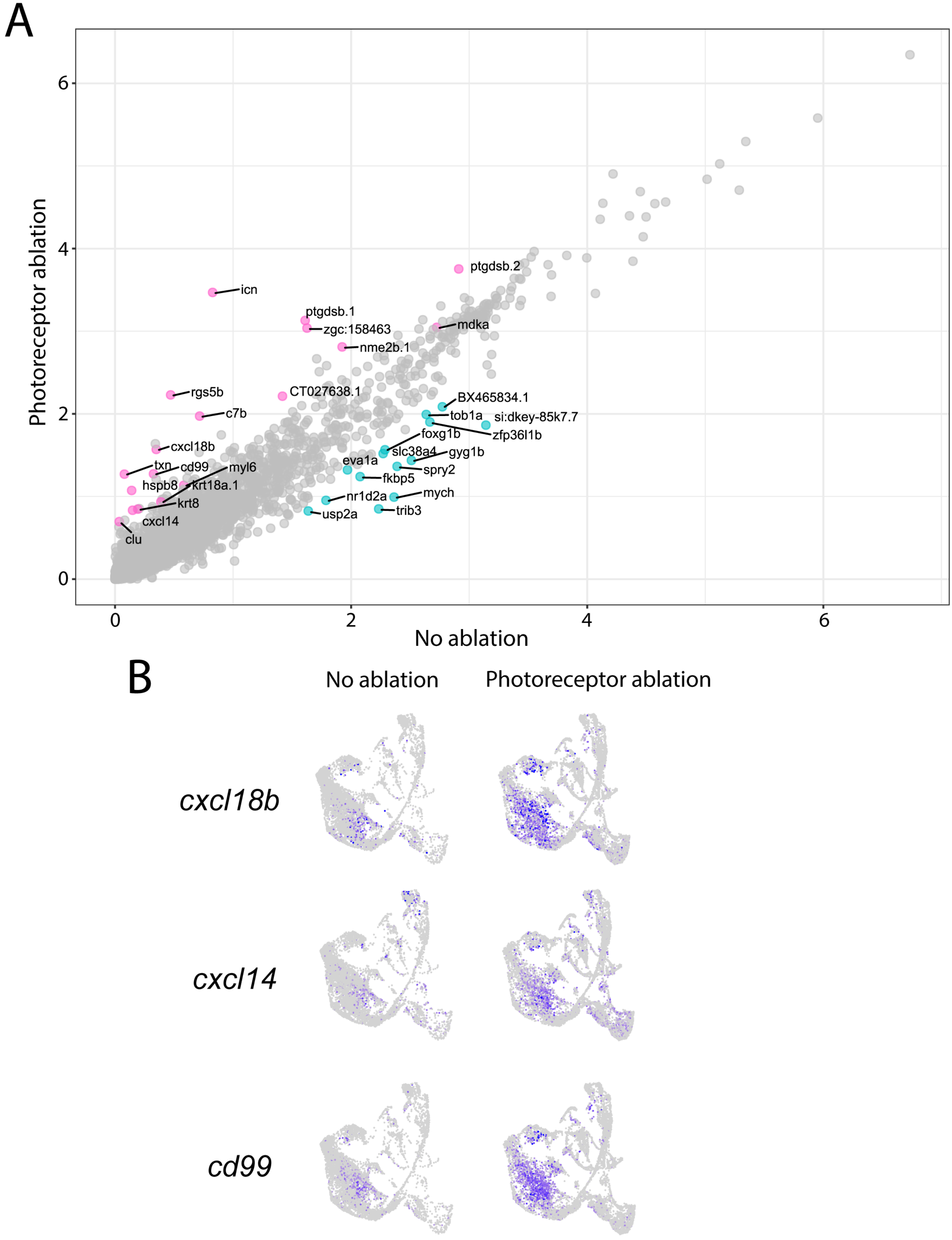

Supplementary Figure 9. (A) Scatterplot indicating genes that are upregulated in resting Müller glia as a result of Lws2 ablation (photoreceptor ablation; pink) or reduced compared to Müller glia in uninjured (no ablation) retinas (cyan). Gene expression values are presented as log1p() expression values, averaged from all cells within the four quiescent Müller glia clusters highlighted in Figure 3. (B) Feature expression plots of genes cxcl18b, cxcl14, and cd99, in Müller glia from the uninjured (no ablation) and Lws2 ablation (photoreceptor ablation) datasets, split by condition.
