# Supplementary material for: Heterogeneity in quiescent Müller glia in the uninjured zebrafish retina drive differential responses following photoreceptor ablation
Source: Front Mol Neurosci. 2023 Jul 27;16:1087136. doi: 10.3389/fnmol.2023.1087136 (PMC10413128; doi:10.3389/fnmol.2023.1087136)
Supplement: Supplementary file 4 [file Image_4.pdf]

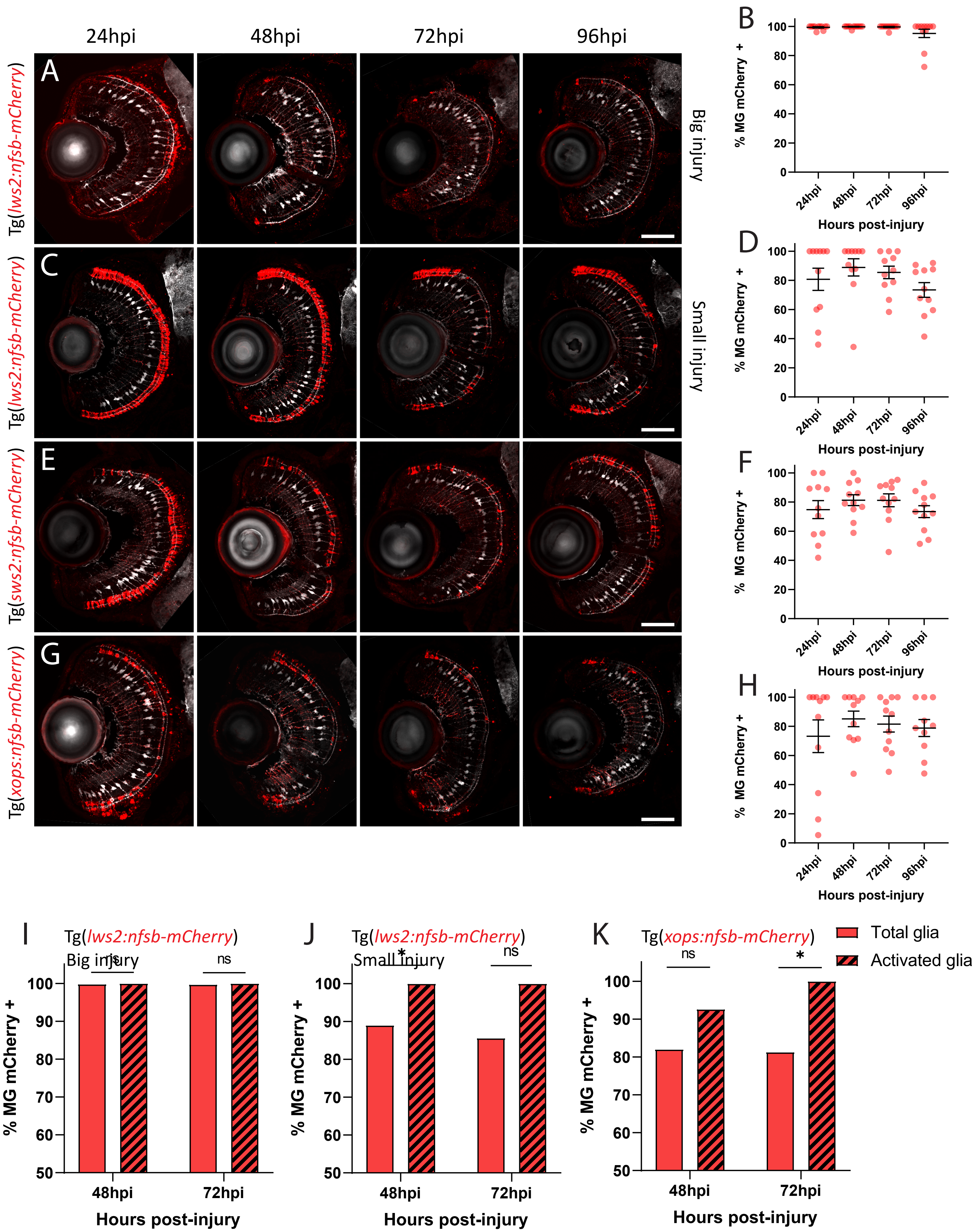

Supplementary Figure 4: (A, C, E, G) Labelling for mCherry antibody reveals presence of photoreceptor debris (red) in Gfap-expressing Müller glia (grey) across injury models from 24-96 hours post injury (hpi). Scale bar = 50 µm. (B, D, F, H) Percentages of Müller glia containing mCherry photoreceptor debris. Cell counts represented as percentages of debris-containing Müller glia between activated and total glia cohorts following widespread Lws2 (I), reduced Lws2 (J) and Xops (K) ablation paradigms. * $p \leq 0.05$.
